# PISTILLATA paralogs in *Tarenaya hassleriana* have diverged in interaction specificity

**DOI:** 10.1186/s12870-018-1574-0

**Published:** 2018-12-22

**Authors:** Suzanne de Bruijn, Tao Zhao, Jose M. Muiño, Eric M. Schranz, Gerco C. Angenent, Kerstin Kaufmann

**Affiliations:** 1 Laboratory of Molecular Biology, Wageningen University, Droevendaalsesteeg 1, 6708 PB Wageningen, The Netherlands; 2 Bioscience, Wageningen Plant Research, Droevendaalsesteeg 1, 6708 PB Wageningen, The Netherlands; 3 Biosystematics Group, Wageningen University, Droevendaalsesteeg 1, 6708 PB Wageningen, The Netherlands; 4 Institute for Biology, Systems Biology of Gene Regulation, Humboldt-Universität zu Berlin, Berlin, Germany; 5 Institute for Biology, Plant Cell and Molecular Biology, Humboldt-Universität zu Berlin, Philippstraße 13, 10115 Berlin, Germany

**Keywords:** PISTILLATA, Flower development, Gene duplications, Paralogs, MADS, Tarenaya, Cleomaceae

## Abstract

**Background:**

Floral organs are specified by MADS-domain transcription factors that act in a combinatorial manner, as summarized in the (A)BCE model. However, this evolutionarily conserved model is in contrast to a remarkable amount of morphological diversity in flowers. One of the mechanisms suggested to contribute to this diversity is duplication of floral MADS-domain transcription factors. Although gene duplication is often followed by loss of one of the copies, sometimes both copies are retained. If both copies are retained they will initially be redundant, providing freedom for one of the paralogs to change function. Here, we examine the evolutionary fate and functional consequences of a transposition event at the base of the Brassicales that resulted in the duplication of the floral regulator *PISTILLATA* (*PI*), using *Tarenaya hassleriana* (Cleomaceae) as a model system.

**Results:**

The transposition of a genomic region containing a *PI* gene led to two paralogs which are located at different positions in the genome. The original *PI* copy is syntenic in position with most angiosperms, whereas the transposed copy is syntenic with the *PI* genes in Brassicaceae. The two *PI* paralogs of *T. hassleriana* have very similar expression patterns. However, they may have diverged in function, as only one of these PI proteins was able to act heterologously in the first whorl of *A. thaliana* flowers. We also observed differences in protein complex formation between the two paralogs, and the two paralogs exhibit subtle differences in DNA-binding specificity. Sequence analysis indicates that most of the protein sequence divergence between the two *T. hassleriana* paralogs emerged in a common ancestor of the Cleomaceae and the Brassicaceae.

**Conclusions:**

We found that the *PI* paralogs in *T. hassleriana* have similar expression patterns, but may have diverged at the level of protein function. Data suggest that most protein sequence divergence occurred rapidly, prior to the origin of the Brassicaceae and Cleomaceae. It is tempting to speculate that the interaction specificities of the Brassicaceae-specific PI proteins are different compared to the PI found in other angiosperms. This could lead to PI regulating partly different genes in the Brassicaceae, and ultimately might result in change floral in morphology.

**Electronic supplementary material:**

The online version of this article (10.1186/s12870-018-1574-0) contains supplementary material, which is available to authorized users.

## Background

Gene duplication can give rise to evolutionary novelty. Selection pressure is temporarily weaker after a duplication event, allowing one or both of the duplicates to evolve in function. Following a gene duplication event, there are several scenarios for the fate of the newly obtained paralogs. Often, one of the paralogs is quickly lost [1]. In case that both paralogs are retained, they might either divide the original function between the two paralogs (subfunctionalization) and/or obtain new functions (neofunctionalization) [2, 3]. Different molecular mechanisms have been proposed to explain how this is achieved [4, 5]. These different mechanisms are not mutually exclusive, and often several mechanisms act on the two paralogs simultaneously or consecutively [6, 7].

In plants, whole genome duplication (WGD) is a common phenomenon, and all angiosperms have undergone at least one WGD [8, 9]. WGDs are implied as a driving force behind the dramatic increase in the number of plant species [10, 11]. Crucially, several key innovations, such as seeds and flowers, coincided with WGDs [12–14]. Interestingly, gene loss after WGDs is not uniform, with some classes of genes being preferentially retained, among which are genes encoding transcription factors (TFs) [5, 15, 16]. One example is the family of MADS-domain TFs [17, 18]. Members of this TF family are involved in virtually all stages of plant development [19] and are well-known for their crucial roles in flower development [20]. They specify the identities of the four different floral organ types in a combinatorial manner according to the (A)BCE model [20–22]. Mechanistically, these TFs achieve this by binding to the promoters of their target genes as organ-specific tetrameric protein complexes, as proposed in the floral quartet model [23].

Based on mutant phenotypes and expression patterns, the functions of A-, B-, C- and E-class proteins are largely conserved throughout the angiosperms [19]. However, many plant lineages retained multiple copies of these genes after duplication events [24–26]. The first floral MADS-box gene paralogs studied in detail were the *Antirrhinum majus* genes *PLENA* (*PLE*) and *FARINELLI* (*FAR*). Whereas *PLE* is the C-function gene in *Antirrhinum,* mutations in the closely related *FAR* gene, surprisingly, only resulted in plants with partial male sterility [27, 28]. *FAR* is partially redundant with *PLE*. However, mutations in these genes result in different mutant plant phenotypes, and the proteins exhibited different capabilities to homeotically specify floral organs, caused by differences in protein-protein interactions [29]. These data suggest that *PLE* and *FAR* have subfunctionalized [28]. *Arabidopsis* also retained paralogous pairs from the C-, as well as the A- and E-classes of MADS-box genes. The paralogous gene pairs show different degrees of divergence. The paralogs of the C-class gene *AGAMOUS (AG)*, the *SHATTERPROOF*s (*SHP1* and *2*), are not involved in floral organ specification, but play a role in carpel and fruit development [30]. In contrast, the four *SEPALLATA* paralogs (*SEP1–4*, E-class) act in a largely redundant manner [31, 32].

The B-function is fulfilled by two genes in eudicot model species - *APETALA3* (*AP3*) and *PISTILLATA* (*PI*) in *Arabidopsis thaliana* [33, 34]*,* and *DEFICIENS* (*DEF*) and *GLOBOSA* (*GLO*) in *Antirrhinum* [35, 36]. The *AP3/DEF* and *PI/GLO* gene lineages resulted from a duplication before the origin of the angiosperms. Within angiosperms, both *AP3*- and *PI*-lineages underwent additional duplications, for example close to the origin of core eudicots [24]. Although *A. thaliana* has only one copy of each B-class gene lineage, other plant species have retained paralogs after these older duplication events [24, 37, 38]. For example, all Solanaceae species have two AP3-like genes (*AP3* and *TM6*), as well as two *GLO* paralogs of more recent origin. These paralogs have subfunctionalized in a partly species-specific manner [39–41]. A similar pattern is seen in basal asterids, where a duplication led to two *PI* paralogs that show species-specific differences in expression patterns [38].

B-class genes do not only specify petal and stamen identity but can also be involved in determining the morphology of these organs. For instance, in *Petunia hybrida* a *PI* paralog is required for the fusion of stamens to the corolla tube [41]. Another example of involvement of B-class genes in morphology is provided by orchids. Orchids possess a perianth that consists of three morphologically distinct types of tepals, and it has been shown that these different tepal morphologies are specified by different combinations of the three to four *DEF* paralogs that are present in orchids [42, 43]. B-class genes might even be able to specify novel floral organ types that are only observed in one species or genus. An example is presented by *Aquilegia*, a basal eudicot that displays an additional type of organ in a whorl between the stamens and carpels, called the staminodium. The specification of this new organ in *Aquilegia* is linked to duplications in the *AP3* lineage [44, 45].

The position of a gene within the genome can be biologically relevant, as genes are dependent on their genomic context for expression. Gene expression is regulated by *cis*-regulatory elements (CREs), which can be dispersed over long distances, even spanning several genes [46]. Epigenetic marks also play a role in regulating gene expression, and these can be different for paralogs located in different parts of the genome. Indeed, in humans it has been found that histone modifications can differ between the original sequence and the copy for segmental duplications [47]. Interestingly, several studies have shown that after gene duplication, the original gene is more evolutionary constrained in sequence than the copy [48]. For these reasons, exceptionally strong conservation of gene order could indicate that the genomic context of a gene is important for its function and/or regulation [49].

This preservation of gene order in different species is called synteny [50–52]. Synteny can be maintained across hundreds of millions of years, e.g. with 90% of the genome being syntenic between human and mice (their ancestral species diverged 90 million years ago (MYA)) [53]. However, in plants synteny is generally less conserved than in animals. This is due to the fact that several rounds of WGD have occurred in plants, and the subsequent process of gene loss and genome rearrangements has blurred syntenic relationships [51, 54, 55]. Still, extensive genome collinearity can be found between closely related species, and plant species that are more distantly related show microsynteny of small genomic regions (of several genes) [55–57].

In comparative genomics, synteny is used to distinguish true orthologs from other homologous genes. Therefore, synteny can provide information about the evolution of gene families. One family for which synteny analysis has helped unravel its evolutionary history is the family of MADS-box genes [58, 59]. One MADS-box gene that displays conserved synteny is the floral B-class gene *PISTILLATA* (*PI*). The synteny of this gene is retained between the sister species of all angiosperms, *Amborella* and almost all other angiosperm species, with the notable exception of the Brassicaceae family [60].

*Tarenaya hassleriana* belongs to the Cleomaceae, which is a sister family to the Brassicaceae [61]. *T. hassleriana* is interesting for comparative studies, as the genome is available, and this species diverged from the Brassicaceae relatively recently (35 million years ago) [62, 63]. This means that *T. hassleriana* is relatively closely related to the well-established model species *A. thaliana*. In contrast to the stereotypic decussate organ arrangement in Brassicaceae flowers, Cleomaceae species exhibit quite diverse floral morphologies [64]. *T. hassleriana’s* basic floral bauplan (4 sepals, 4 petals, 6 stamens and 2 fused carpels) is similar to *A. thaliana’s*, but in contrast to the disymmetric Arabidopsis flowers, the flowers of Cleomaceae species display bilateral symmetry [64]. *T. hassleriana* has two paralogs of the B-function gene *PI* [65]. These *PI* paralogs are probably derived from the At-β-duplication at the origin of the Brassicales, ca. 70 MYA [66].

We questioned whether the genomic location of *PI* could influence its function, as the synteny of *PI* is conserved throughout the angiosperms, with the exception of the Brassicaceae. *T. hassleriana* has two *PI* paralogs, and being closely related to *A. thaliana*, may have traits that are intermediate between the Brassicaceae and other eudicots. Here, we investigated how these two *PI* paralogs in *T. hassleriana* diverged from each other, focusing on expression patterns and several functional features of the two TFs. Our data indicate that both PI paralogs have similar expression patterns, but diverged from each other in their biochemical properties, which could imply divergence in gene function. This finding has interesting implications for the functional evolution of *PI* genes in the Brassicaceae.

## Results

### Phylogenetic analysis shows that one of the Tarenaya *PI* paralogs clusters with the Brassicaceae *PI* genes

**Fig. 1:**
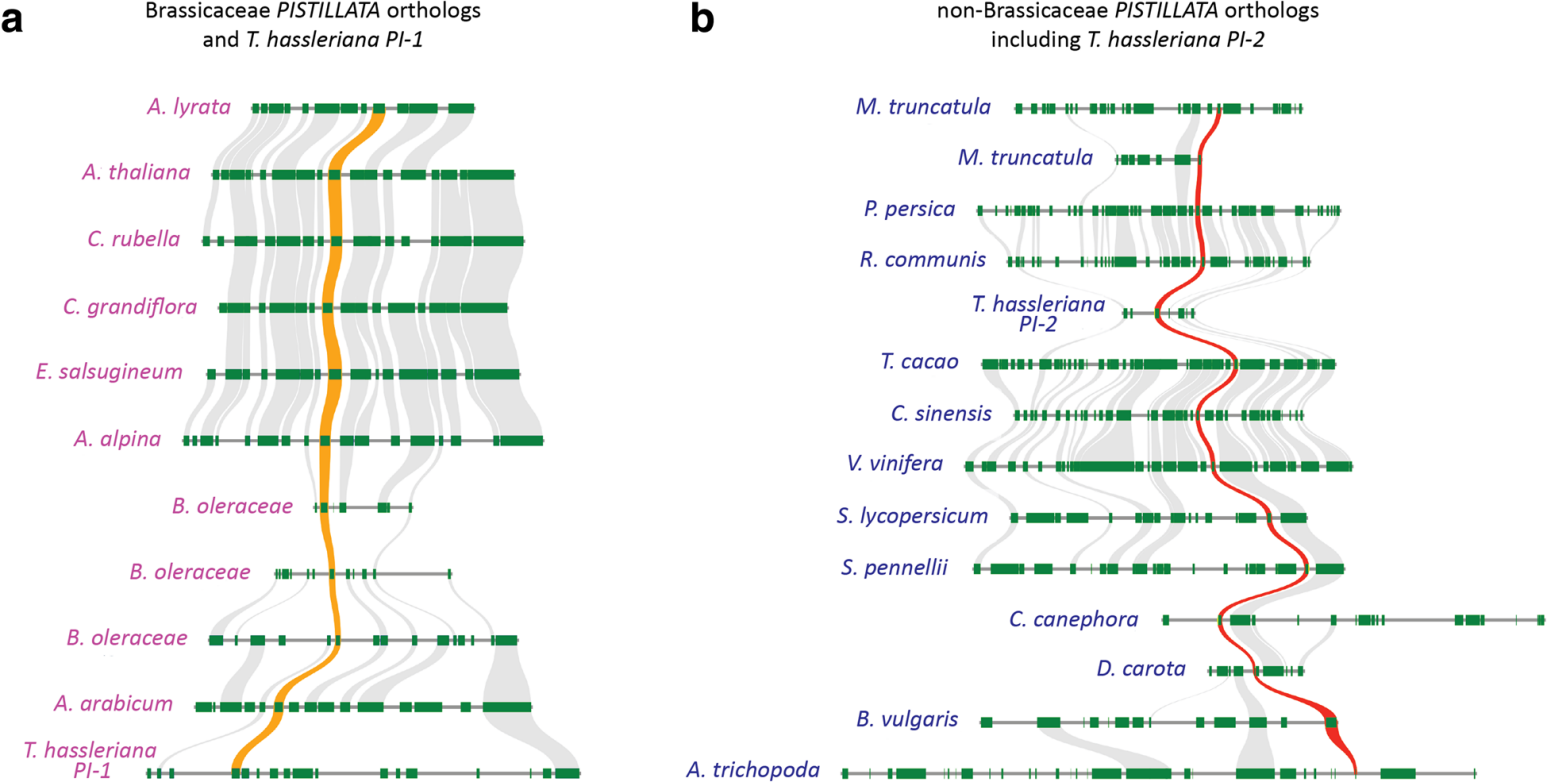
Synteny of both *PI* paralogs of *T. hassleriana*. *PI* paralogs are shown with an orange (Brassicaceae) or red (non-Brassicaeae) trace. Other syntenic genes are linked in grey. **a** Synteny of *ThPI-1* with the Brassicaceae *PI* orthologs. Shown are the Brassicaceae type I genera Arabidopsis (*A. lyrata* and *A. thaliana*) and Capsella (*C. rubella* and *C. grandiflora*); the Brassicaceae type II species *Eutrema salsugineum*, *Arabis alpina*, *Brassica oleraceae* (3 paralogs), the basal Brassicaceae *Aethionema arabicum* and *PI-1* of *T. hassleriana*. **b** Synteny of *ThPI-2* with non-Brassicaceae angiosperms. Shown are 7 rosid species (*Medicago truncatula* (2 paralogs), *Prunus persica*, *Ricinus communis*, *T. hassleriana*, *Theobroma cacao*, *Citrus sinensis* and *Vitis vinifera*), 3 asterid species (*Solanum lycopersicum*, *Solanum pennellii* and *Coffea canephora*), one Caryophyllales (*Beta vulgaris*) and the sister species to all other angiosperms, Amborella (*A. trichopoda*)

**Fig. 2:**
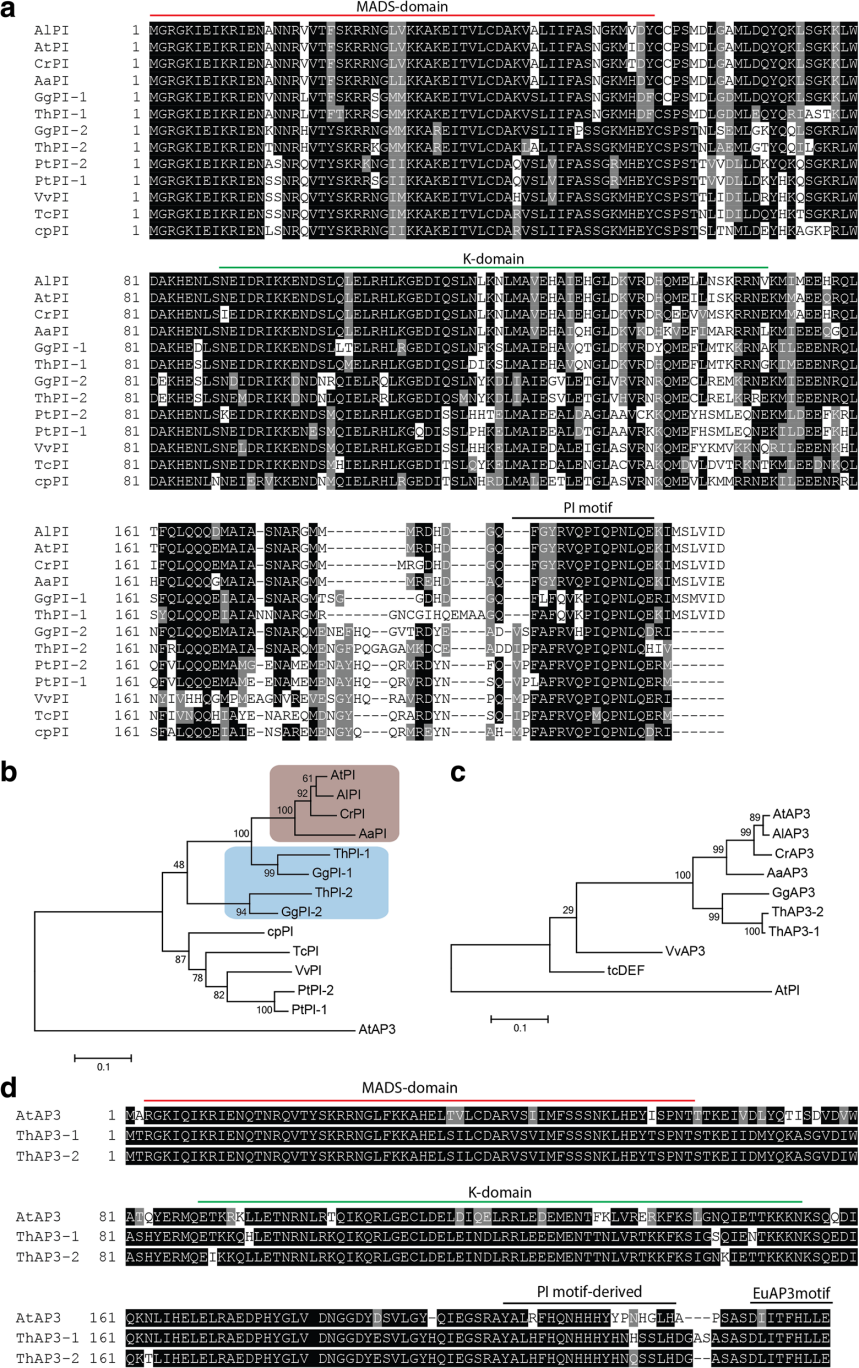
Phylogenetic and sequence analysis of the *T. hassleriana* B-class genes. **a** Alignment of PI orthologs from several species. **b** Maximum-likelihood phylogeny of PI orthologs. The PI orthologs belonging to Brassicaceae species are indicated in beige, the Cleomaceae PI orthologs in blue. **c** Maximum-likelihood phylogeny showing the position of the ThAP3 paralogs. Alignments for phylogenies were generated with a codon-based DNA-sequence algorithm. **d** Alignment of ThAP3 paralogs with AtAP3. The MADS-domain, the K-domain and the lineage-specific C-terminal motifs are indicated in A and D. Abbreviations: At = *A. thaliana*; Al = *A. lyrata*; Cr = *Capsella rubella*; Aa =  *Aethionema arabicum*; Th = *T. hassleriana*; Gg = *G. gynandropsis*; Cp = *Carica papaya*; Tc = *Theobroma cacao*; Vv = *Vitis vinifera*; Pt = *Populus trichocarpa*

Previously, it was found that *T. hassleriana* possesses two copies of each B-class gene, *APETALA3* and *PISTILLATA*. Interestingly, the *PI* paralogs are in different genomic locations. One of the *ThPI* paralogs shares conserved synteny with the Brassicaceae-specific *PIs* (*ThPI-1*), whereas the other *PI* paralog (*ThPI-2*) is syntenic with the other eudicots (Fig. 1) [65]. ThPI-1 (215 AA) and ThPI-2 (214 AA) are highly divergent in sequence, sharing only 62% protein sequence identity (68% at the nucleotide level) (Fig. 2a). Here we present a more detailed phylogeny of *PI* (Fig. 2b), which shows that *ThPI-1* that is syntenic with the Brassicaceae clearly clusters with Brassicaceae *PI*-clade genes. The other paralog, *ThPI-2,* is less closely related to this group. Phylogeny reconstructions performed by the Maximum-likelihood method (Fig. 2b) and the Neighbour-Joining algorithm produced similar results (not shown). *ThPI-1* clusters with Brassicaceae, indicating that *ThPI-1* resembles the Brassicaceae *PI* genes in sequence. This means that a substantial part of the sequence divergence observed between *ThPI-1* and *ThPI-2* arose in *ThPI-1* before the split between the Brassicaceae and Cleomaceae. This is also reflected in specific patterns of sequence divergence in the C-terminal part of the sequence. It has been shown that the PI motif, which is located in the C-terminus (see Fig. 2a), contains signature amino acids able to distinguish between the PI lineages from Brassicaceae and other eudicots [67]. Like the Brassicaceae PI orthologs, ThPI-1 (but not ThPI-2) misses the first 2 AA of the PI motif. ThPI-1 (but not ThPI-2) also resembles the Brassicaceae PI proteins in having a C-terminal extension of six amino acids compared to other eudicot PIs [67]. Once again, this indicates that these changes to the PI protein sequence occurred before the Brassicaceae-Cleomaceae split, when there were two paralogs of PI present in the genome of the common ancestor of these families. Interestingly, both *PI* paralogs are also retained in the Cleomaceae species *Gynandropsis gynandra*, hinting that both copies might be functional (Fig. 2a, b). In contrast, the two *AP3* paralogs of *T. hassleriana* originate from a tandem duplication that is thought to be recent [65], as the two paralogs are highly similar (Fig. 2c, d, Additional file 1: Figure S1). We found that this tandem duplication is not present in *G. gynandra,* which supports the idea that this tandem duplication occured recently, since *Gynandropsis* and *Tarenaya* split no more than 13.7 MYA [68].

### The two *ThPI* paralogs did not diverge in expression pattern

It is known that the genomic context of a gene may influence its expression [69, 70]. As the *T. hassleriana PI* paralogs have different genomic environments, we investigated whether they diverged from each other in expression pattern. It was previously shown that both *PI* paralogs in *T. hassleriana* are expressed during flower development. More specifically, RT-qPCR data showed that these genes are expressed differentially during flower development, as well as in mature petals and stamens [65]. Here, we investigated the expression patterns of these genes in more detail, using RNA in situ hybridization.

We designed probes for *ThAP3*, *ThPI-1* and *ThPI-2.* The *ThAP3* probe cross-hybridizes with transcripts from both *ThAP3* paralogs, since the high similarity between these genes did not allow for the design of specific probes (see Additional file 1: Figure S1). The *ThAP3* probe covered part of sequence encoding the K-domain and the C-terminus, as well as the 3’ UTR. The probes for the *PI* paralogs only covered the C-terminal part of the mRNA and the 3’UTR (which we determined using 3’RACE), not the region coding for the K-domain. Although these two *PI* probes share only 65% similarity at nucleotide level (longest continuous stretch of identical sequence is 14 bp), we cannot exclude some cross-hybridization with mRNA from the other paralog.

**Fig. 3:**
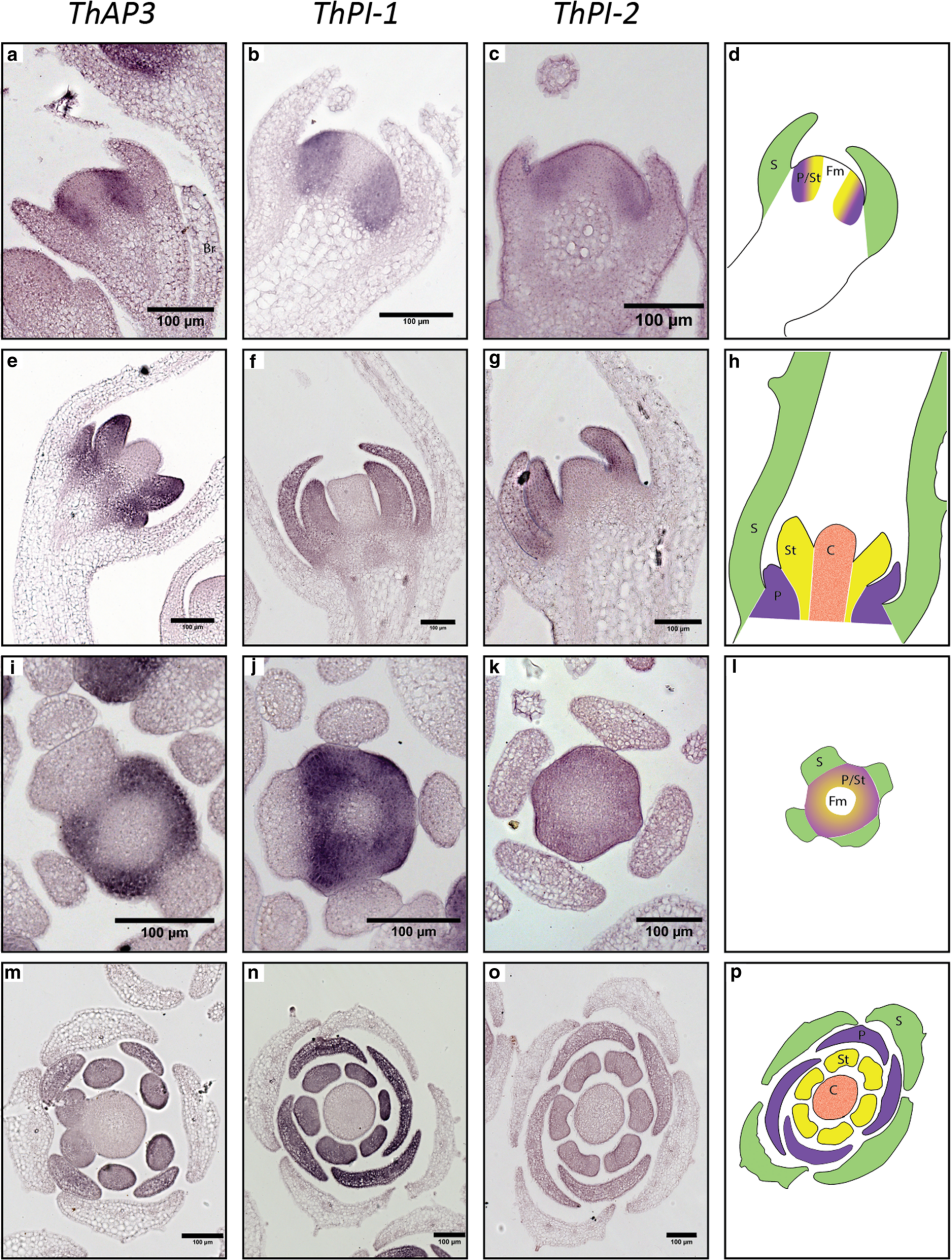
Expression patterns of *T. hassleriana* B-class genes. Expression patterns of the *ThAP3* paralogs (**a**, **e**, **i**, **m**), *ThPI-1* (**b**, **f**, **j**, **n**) and *ThPI-2* (**c**, **g**, **k**, **o**) as determined by RNA in situ hybridization. Expression was determined in early developmental stages before organ primordia were formed (**a**-**c**, **i**-**k**) as well as later during organ differentiation (**e**-**g**, **m**-**o**). Schematics of the different developmental stages and planes are shown in **d**, **h**, (longitudinal),L and P, (cross). Br=bract; S=Sepal primordia (green); P/St = whorls giving rise to petals and stamens; P = petal primordia (purple); St = stamen primordia (yellow); C = carpel primordia (orange); Fm = floral meristem. Scale bar = 100 μm

**Fig. 4:**
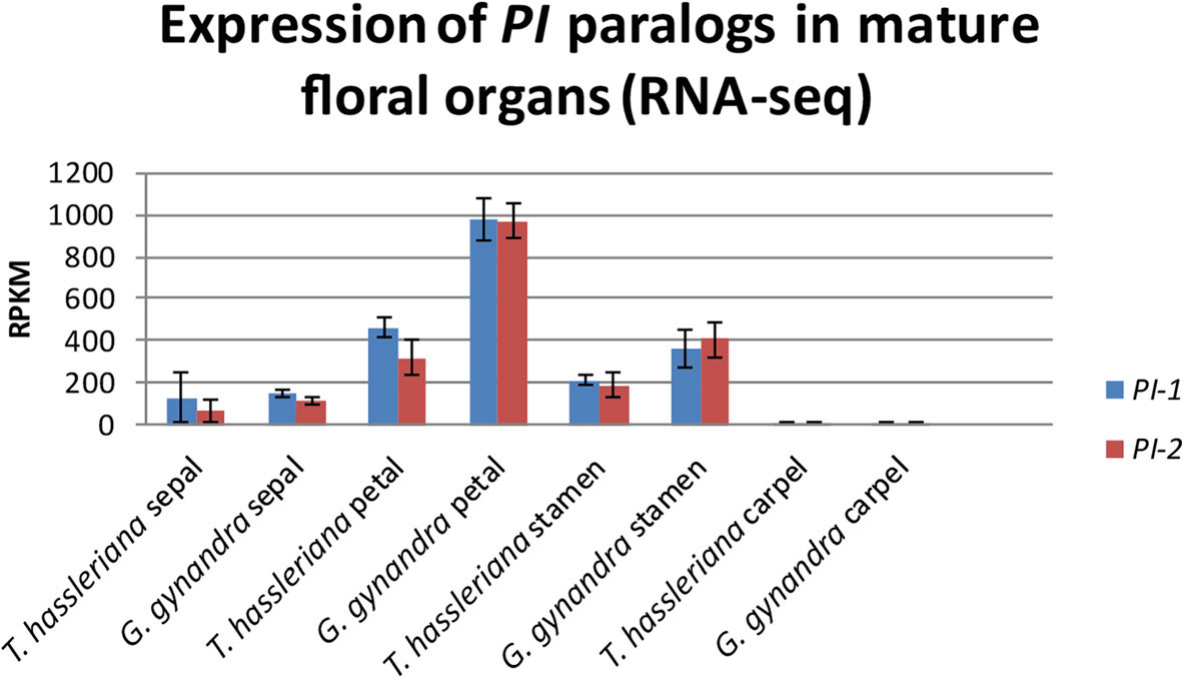
Expression levels of *ThPI-1* and *ThPI-2*, according to RNA-seq data of mature floral organs. Error bars represent standard deviation. RPKM = reads per kilobase of transcript per million mapped reads. RNA-seq data obtained from [72]

Early during development, when only sepal primordia are present (comparable to *A. thaliana* floral stage 3–4 [71]), *ThAP3* was found to be expressed in the meristematic cells that will give rise to whorls 2 and 3 (Fig. 3a and i), and at later developmental stages, when primordia of all organs become visible, expression of *AP3* is specific to petal and stamen primordia (Fig. 3e and m). The expression patterns of *ThPI-1* and *ThPI-2* closely resemble each other. During early stages, expression of both genes can be seen in cells that will give rise to whorls 2 and 3 (Fig. 3b, c, j and k). Later during development, expression is seen in developing petal and stamen primordia (Fig. 3f, g, n and o). A previous study showed quantitative differences in expression level between the two paralogs during development and in mature floral organs [65]. However, our data show that *ThPI-1* and *ThPI-2* are both expressed in petals and stamens, with no detectable differences in expression pattern between the two paralogs. It seems therefore that both *PI* paralogs did not diverge in spatial and temporal expression patterns, at least during the analyzed developmental stages. Interestingly, an RNA-seq experiment on mature floral organs did not show significant differences in expression levels between the two paralogs (Fig. 4, data from [72]).

### Heterologous expression of *Th*PI paralogs in *A. thaliana* results in different phenotypes

**Fig. 5:**
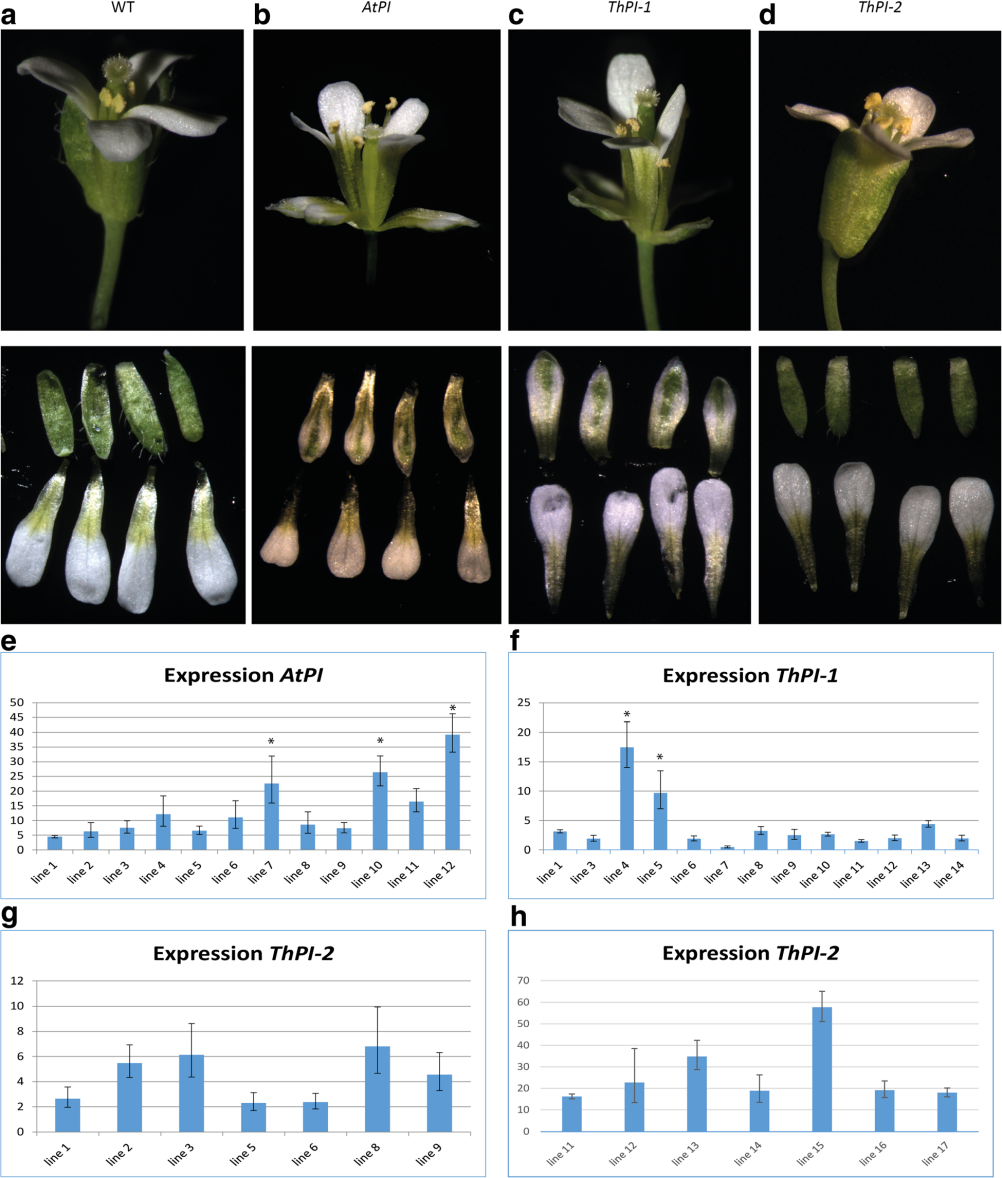
Heterologous expression of the *ThPI* paralogs in *A. thaliana*. **a** a wildtype (WT) *A. thaliana* flower. **b** a *35S::AtPI* flower, which shows the phenotype obtained by constitutive expression of the native *PI*. Note the change in orientation of the first whorl organs. **c** a *35S::ThPI-1* flower, showing homeotic conversion of sepals to petals. **d** a *35S::ThPI-2* flower, showing no aberrant phenotype. Top row shows whole flower, bottom row shows dissected sepals (top) and petals (bottom). **e** *AtPI* expression levels in *35S::AtPI* lines. **f** *ThPI-1* expression levels in *35S::ThPI-1* lines. **g** *ThPI-2* expression levels in *35S::ThPI-2* lines. **h** *ThPI-2* expression levels in additional *35S::ThPI-2* lines. Lines that showed an overexpression phenotype are indicated with an asterisk. Expression measured in leaves, and calculated relative to a reference gene (*TIP41*). For the expression in **e**, **f** and **g**, Rosette leaves of 6–8 week plants were used. In **h**, the first developing rosette leaves were used

Although the two *ThPI* paralogs do not appear to have diverged in expression pattern, they did substantially diverge in protein sequence. We therefore hypothesized that some of their protein functions might have changed during evolution. As a first test for protein function, we expressed both *ThPI* paralogs constitutively in wildtype *A. thaliana*. As a control, we also created lines with *AtPI* overexpression. Constitutive overexpression of the native *PI* in *A. thaliana* has been reported to lead to partial conversion of sepals to petals, due to low expression of *AP3* in the outer whorl [73]. We analyzed twelve *35S::AtPI* lines, and obtained sepal to petal conversion in 3 independent lines (Fig. 5b). Expression analyses indicated that lines showing a modified phenotype had the highest level of transgene expression (Fig. 5e). For *ThPI-1*, the paralog that is most similar to the Brassicacae *PI*, the 2 lines with the highest transgene expression level (out of 13 lines) displayed partial homeotic conversions of sepals into petaloid organs, similar to the *35S::AtPI* lines (Fig. 5c, f). This indicates that ThPI-1 is capable of performing similar functions as AtPI in the first whorl of *A. thaliana*. For ThPI-2*,* although we analyzed 14 *35S::ThPI-2* lines, we did not observe an aberrant phenotype in any of these lines (Fig. 5d, g, h). This could indicate that ThPI-2 is unable to specify petals in the first whorl of *A. thaliana*, suggesting that ThPI-2 is functionally different from AtPI. Thus, the two *T. hassleriana* proteins appear to be biochemically different.

### The ThPI paralogs differ in their ability to form protein-protein interactions

The heterologous expression assay indicates that the *ThPI* paralogs might functionally differ. As MADS-domain TFs function as part of protein complexes [23, 74, 75], we tested whether the two ThPI paralogs have different capabilities to form protein complexes. Differences in protein complex formation can be relevant, as divergence in protein-protein interactions may lead to divergent gene regulation. TFs need to bind DNA to exert their functions, therefore DNA-binding protein complexes were analyzed using Electrophoretic Mobility Shift Assays (EMSAs), a well-established method to study DNA-binding of MADS-domain protein-complexes [36, 76].

**Fig. 6:**
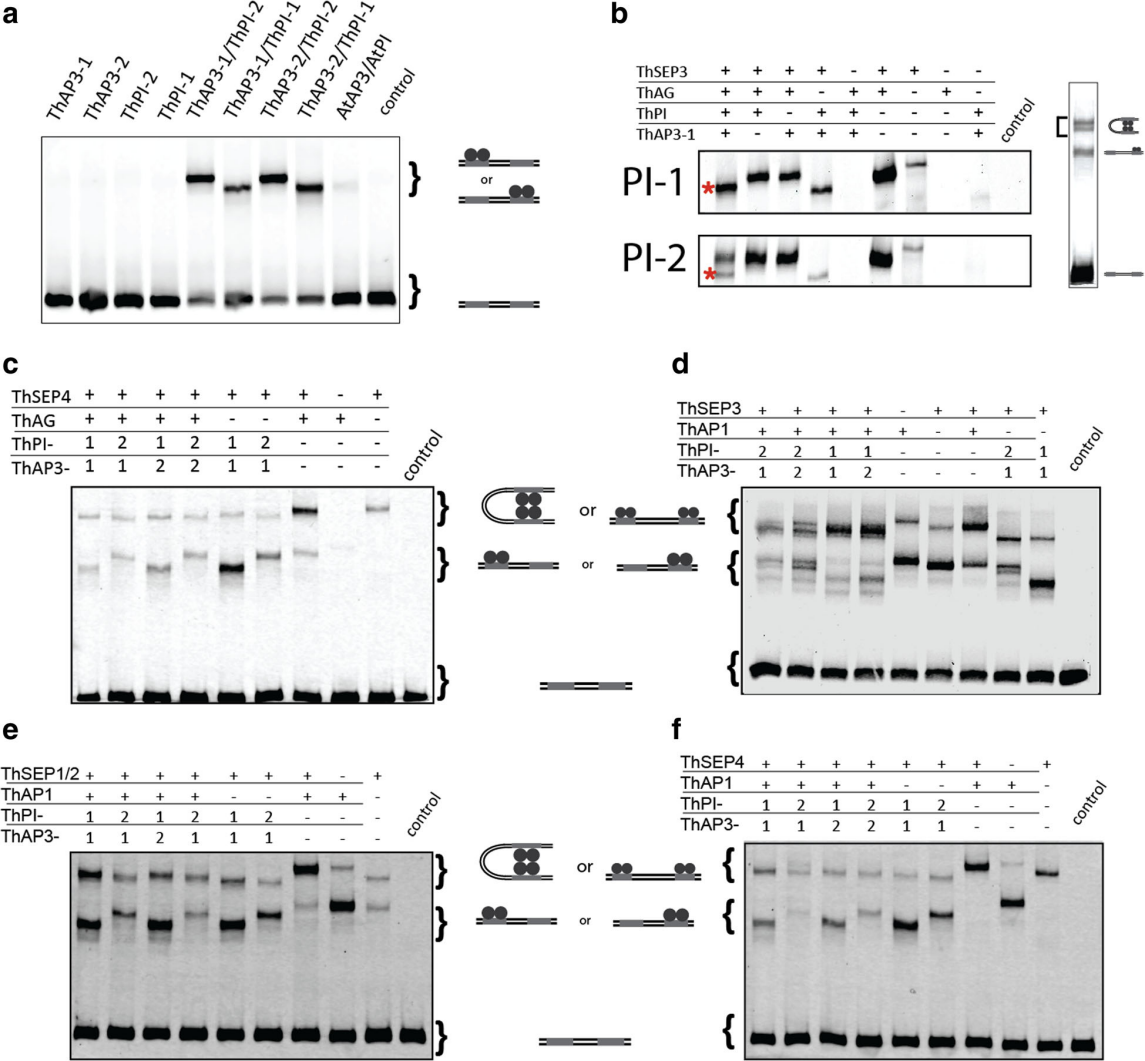
DNA-binding protein-complexes formed by *T. hassleriana* B-class proteins. In **(a)**, homo- and heterodimerization of ThAP3 and ThPI **(b)** Complexes formed with ThAG, ThSEP3 (Th01528), ThAP3–1 and either of the two ThPI paralogs. The figure shows only the higher order complexes (tetramers); in the right image this part compared to the whole gel is indicated. In **(c)**, EMSA for protein complexes with ThAG and a ThSEP4 paralog (Th21984). **(d, e, f)** EMSAs testing the formation of a DNA-binding protein complex of the B-class dimers with a ThAP1 paralog (Th13754) and with different ThSEP paralogs: ThSEP3 (Th1528) (**d)**, ThSEP1/2 (Th2854) (**e)**, and ThSEP4 (Th21984) (**f)**. For all experiments, a promoter fragment from the *A. thaliana* *SEP3* promoter was used as probe (Smaczniak et al., 2012a). The control is an empty-vector control, in which no MADS protein production is expected

Initially, we analyzed interactions between the two ThAP3 and the two ThPI proteins. We could not detect DNA-binding by homodimers of any of the four B-class proteins (Fig. 6a). This is not surprising, as AP3- and PI-type proteins form obligate heterodimers in most eudicots that have been analyzed so far [76–78]. We detected heterodimers for all four possible AP3-PI combinations (Fig. 6a), indicating that there has been no subfunctionalization at the level of protein dimerization. Interestingly, ThPI-2 containing dimers migrate slower through the gel than ThPI-1 containing dimers, even though they have similar molecular mass (24.72 vs 24.95 kDa) and charge status, suggesting a difference in protein conformation (Additional file 2: Table S1).

Although there are no apparent differences between ThPI-1 and ThPI-2 in their ability to form heterodimers with ThAP3 paralogs, it is possible they have different abilities to form larger protein complexes. According to the floral quartet model, B-class proteins act in tetramers with other MADS-domain TFs [23, 79]. To determine whether the ThPI paralogs differ in higher-order complex formation, we investigated their ability to form complexes with members of other homeotic protein classes. According to the floral quartet model, we expect the B-class proteins to interact with a SEPALLATA (SEP) protein and APETALA1 (AP1) in petals, whereas a complex of the B-class proteins with one AGAMOUS (AG) and one SEP protein should specify stamens. *T. hassleriana* has one *AG* gene and two genes each for *SEP1/2*, *SEP3* and *SEP4* [65]. Focusing on the stamen-specific complex, we analyzed whether the ThPI paralogs interact differently with ThAG and the two ThSEP3 paralogs. SEP3 was chosen as a representative SEP protein, as it is suggested to be the most active *A. thaliana* SEP protein based on the number of different protein-interactions it forms [80]. First, we compared higher-order complex formation of all four different B-class heterodimers. As expected, the two ThAP3 paralogs behaved similarly in these experiments (Additional file 3: Figure S2A). However, the two ThPI paralogs showed differences in higher-order complex formation. Whereas one higher-order complex (besides a dimer complex) was observed for combinations containing ThPI-1, two tetrameric complexes were observed when ThPI-2 was present. This pattern was found for both ThSEP3 paralogs (Additional file 3: Figure S2A). We studied the composition of these different complexes in more detail for one of the ThSEP3 paralogs (Th1528) (Fig. 6b, Additional file 3: Figure S2B, C). Using dropout experiments, it could be concluded that the single tetrameric complex observed with ThPI-1 consists of ThAP3/ThPI-1/ThAG/ThSEP3, which is the expected complex for stamen-specification. A similar complex (ThAP3/ThPI-2/ThAG/ThSEP3) was observed with ThPI-2 (Fig. 6b, marked with an asterisk). However, when ThPI-2 is present, a second tetrameric complex (upper band) is observed. This other complex does not contain any B-class proteins, but instead consists of only ThSEP3 and ThAG. The fact that a ThAG/ThSEP3 tetramer is formed in addition to a ThAG/ThSEP3/ThAP3/ThPI-2 complex suggests that a fraction of ThSEP3/ThAG dimers bind to each other, instead of to a ThAP3/ThPI-2 dimer. These data indicate that there are differences between the two ThPIs in affinity to form a higher-order complex with AG/SEP3. The affinity of ThAG/ThSEP3 for ThPI-2 is lower than for ThPI-1, because for ThPI-1 all ThAG/ThSEP3 dimers are incorporated into a ThAG/ThSEP3/ThAP3/ThPI-1 complex.

Summarizing, both ThPI paralogs are capable of forming a complex with ThAG and ThSEP3. However, the data suggest that they do so with different affinities, as ThPI-1 shows a higher affinity for this complex than ThPI-2.

We next investigated whether ThPI-2 has a lower affinity than ThPI-1 to form tetramers in general, or whether it is specific for certain protein combinations. We therefore first analyzed tetramer formation with ThAG and a different ThSEP paralog. Interestingly, a single tetrameric complex was observed for both of the ThPI paralogs when a ThSEP4 paralog (Th21984) was used (Fig. 6c). This indicates that the lower affinity to form a ThAG/ThSEP/ThAP3/ThPI complex with ThPI-2 than with ThPI-1 may not be a general feature of ThPI-2.

Next, we studied combinations of the B-class proteins with one ThSEP and one of the ThAP1 paralogs, a protein complex that is important for petal formation based on data from Arabidopsis (Fig. 6d, e, f). Interestingly, using ThSEP3, we observed a single complex when ThPI-1 is present, whereas two complexes are formed when ThPI-2 is present. Similar to what we observed for combinations with ThAG, it seems that the higher complex observed for combinations with ThPI-2 may not contain the B-class proteins, as it runs at the same height as ThAP1 homotetramers. When we tested the interaction of the B-class paralogs with ThAP1 and ThSEP4, we obtain a single complex for either of the ThPI paralogs, again indicating that there is no difference in complex formation with ThSEP4. When we examined combinations of the B-class proteins with ThAP1 and another SEP, ThSEP1/2, we observed a single complex for each protein combination. However, complexes containing ThPI-1 run at a different height than combinations with ThPI-2. These differences in gel shift indicate that these complexes will likely have a different protein composition, but we did not study these differences in detail.

From these experiments it can be concluded that ThPI-1 and ThPI-2 are biochemically different, as they show differences in their affinities to form higher-order complexes. ThPI-2 has a lower affinity for certain higher order complexes than ThPI-1. However, this does depend on the interaction partners, as different ThSEP paralogs gave different results. We can conclude that the ThPI paralogs (as well as the ThSEP paralogs) are diverged in their ability to form DNA-binding higher order protein complexes.

### DNA-binding specificity

In EMSA experiments, usually a single or only a few DNA probes are used and the interaction of these probes with various protein complexes can be tested. However, it is also possible that the two ThPI paralogs differ in their DNA-binding specificity and/or general affinity to DNA. To analyze this, we used SELEX-seq (Systematic Evolution of Ligands by EXponential enrichment followed by deep sequencing) [81] to test whether there are any differences in DNA-binding specificity between the two ThPI paralogs. We performed SELEX-seq experiments on ThAP3/ThPI heterodimers using a custom-made *A. thaliana* AP3 antibody, which recognizes both *T. hassleriana* AP3 paralogs (see Additional file 4: Figure S3).

**Fig. 7:**
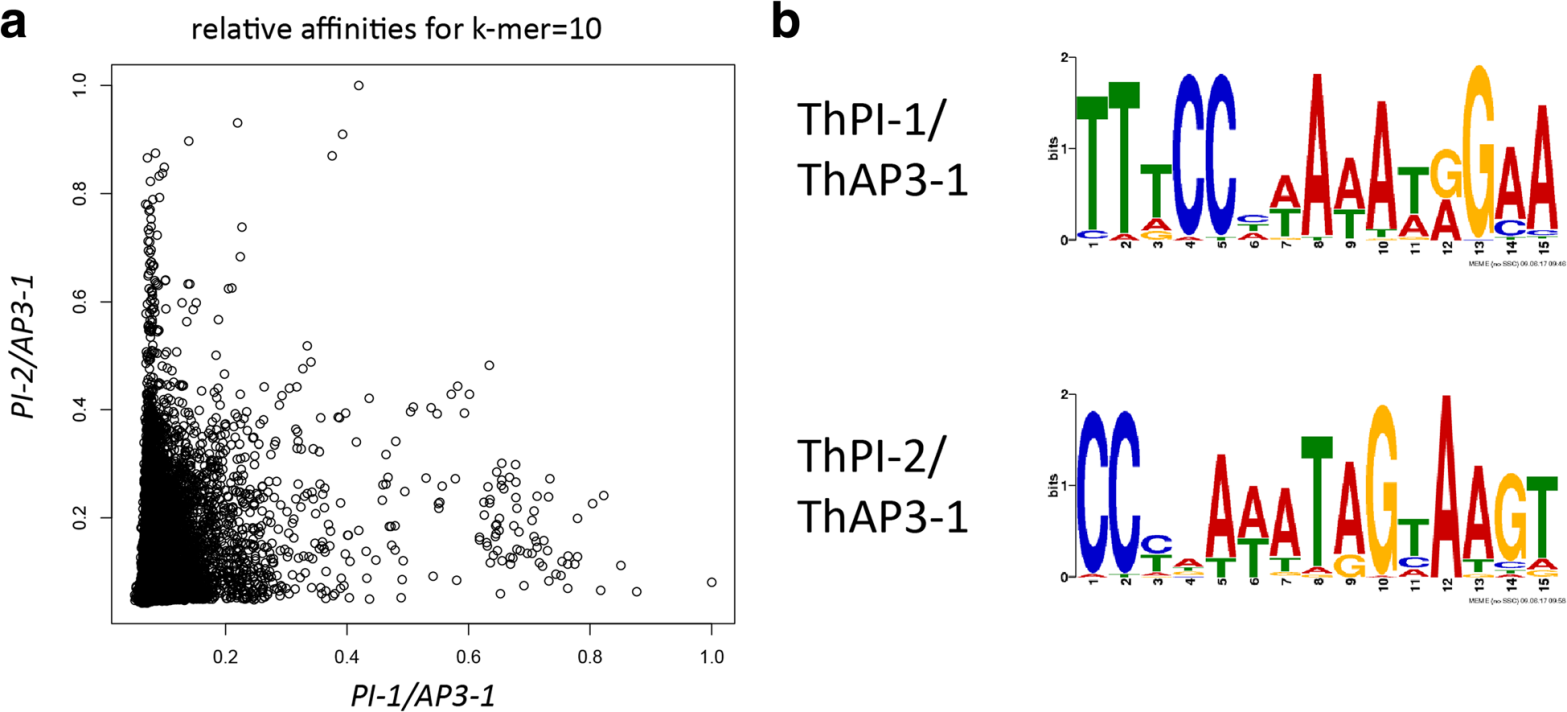
DNA-binding specificities as determined by SELEX-seq. **a** Dotplot comparing the relative affinities between ThPI-1/ThAP3–1 and ThPI-2/ThaP3–1. **b** Motifs obtained for each of the PI/AP3–1 heterodimers. Motif discovery was performed on the most recurring 40 N sequence for each of the 0.1% k-mers with the highest affinity

Good enrichment of bound sequences was obtained for the heterodimers ThPI-1/ThAP3–1 and ThPI-2/ThAP3–1, in SELEX round 8 and 5 respectively, as shown by EMSAs (Additional file 5: Figure S4A, B). We sequenced these SELEX rounds, and obtained ~ 0.3 million reads for PI-1/AP3–1 and ~ 30 million reads for PI-2/AP3–1, with a percentage of perfect CArG-boxes (CC[A/T]_6_GG) of 12,6 and 14% respectively, indicating that we indeed have good enrichment of ThAP3/ThPI bound sequences. We calculated relative affinities of the heterodimers for each possible 10 bp sequence (k-mer), and compared these between the two different ThPI heterodimers. This comparison showed that there are differences in DNA-binding specificity between the two different ThPI proteins (Fig. 7a). For each heterodimer, we performed a motif search using MEME. The motif search was performed for the top 0.1% of k-mers (1035 for ThPI-1, 483 for thPI-2) with the highest affinity, and for each of these k-mers the most recurrent sequence containing this k-mer was used. For both ThPI paralogs we find a motif resembling a CArG-box (Fig. 7b). For each heterodimer, the motif shows two conserved cytosines in the beginning of the motif, whereas at the 3′ end the first guanine is less conserved than the second. However, the motifs are slightly different from each other, in particular the A-rich stretch in the centre. In addition, although both motifs show specific nucleotides in positions neighboring the core CArG-box, these extensions are slightly different, with the extension of the ThPI-1 motif consisting of two adenines, whereas for ThPI-2 this motif is a thymine followed by two adenines. Taken together, these data indicate subtle differences in DNA-binding specificities between the two different ThPI paralogs.

## Discussion

The synteny of the *ThP*I paralogs is interesting: generally, the genomic location of *PI* is conserved throughout the angiosperms. However, the duplication that led to the *ThPI* paralogs transposed one of the *PI* copies into a different genomic location. Whereas *ThPI-2* shares very conserved synteny with *PI* orthologs from the rest of the eudicots, *ThPI-1* is situated in a different genomic location, which it shares with the Brassicaceae.

Although closely related plant species show extensive genome colinearity [55, 56, 82–85], plant species that are more diverged do not show large amounts of synteny conservation. However, microsynteny of small genomic regions (of several genes) can be found between distant plant lineages, with examples found even between rice and Arabidopsis (diverged 200 MYA) [53, 57]. Interestingly, conservation of microsynteny is not uniform over the genome [53, 57, 86]. This might indicate that synteny is more important for certain genomic regions, and possibly certain genes. Both B-class genes show extreme synteny conservation. *PI* is conserved in synteny in most angiosperms, except the Brassicaceae. The Cleomaceae are an intermediate form, having one *PI* paralog that is syntenic with most other angiosperms, and the other one shares its position with the Brassicaceae.

Considering the largely conserved synteny of PI across most angiosperms, the question arises whether the transposition of *ThPI-1* into a new genomic location influenced the regulation or function of the gene.

### *Th*PI paralogs diversified biochemically

The two *Tarenaya hassleriana PI* paralogs share 62% protein identity (duplication 70 million years ago [66]). This amount of sequence divergence falls within the range observed for functional B-class paralogs in other species. For comparison, the GLO paralogs in the Solanaceae have 63–70% protein identity (around 108 million years old [87]), and the paralogs MtPI and MtNGL9 in *Medicago truncatula* share 73% protein identity (duplication occurred around 39 million years ago) [88, 89]. Interestingly, the “original” (*ThPI-2*) and the transposed *PI* (*ThPI-1*) paralog diverged in sequence; the transposed paralog resembles the Brassicaceae *PI* in containing a less conserved PI-motif and a six amino acids extension compared with the *PI* homologs of most other eudicots. It would be interesting to create a detailed phylogeny for *PI* in the Brassicales, analyzing more Cleomaceae and Brassicacae species, as well as species in the other families within the Brassicales. Such a phylogeny could help answering questions about the evolutionary history of *PI*, such as when exactly the transposition took place, when one copy was lost in the Brassicaceae, and whether this copy was lost in more Brassicales families. A more detailed phylogeny could also be used to analyze whether the selection pressure on both paralogs was similar after the duplication.

We investigated if and how the two *T. hassleriana PI* paralogs have diverged from each other. Although based on RT-qPCR data it was reported that these genes differ in their level of expression, with *ThPI-1* being higher expressed [65], our analysis of spatio-temporal expression patterns did not show any differences between the two *ThPI* paralogs, at least in the analyzed developmental stages. In addition, we analysed RNA-seq data of mature floral organs from another study, in which we did not find significant differences in expression levels between the two paralogs [72]. Therefore, it remains unclear whether these genes differ in their level of expression. While the *ThPI* paralogs did not subfunctionalize in spatiotemporal expression pattern, we found that the observed sequence divergence has led to functional differences between the two proteins. In our heterologous expression experiment in *A. thaliana*, only ThPI-1, but not ThPI-2, was able to homeotically transform sepals into petaloid structures. This indicates that the proteins may differ in function, although this was only tested in a heterologous system so far and we cannot exclude that the transgene expression levels in our 14 *35::ThPI-2* lines were not high enough to induce a phenotype in the first whorl organs (Fig. 5g, h), even though we analyzed a similar number of lines for *35S::ThPI-2* as we did for *35S::AtPI* and *35S::ThPI-1*. Subsequently, we performed two in vitro assays to determine whether the protein properties are different: EMSA to determine differences in the formation of DNA-binding protein complexes, and SELEX-seq to investigate the DNA binding specificities. The EMSA results show that all four possible AP3-PI heterodimers are formed in vitro, indicating that there has been no subfunctionalization at the level of protein dimerization. Subfunctionalization at the dimerization level has been observed for B-class paralogs in some species [40, 90], but not in others [89, 91, 92]. As the ThAP3 paralogs are highly similar to each other, it is not surprising that we did not find subfunctionalization at the dimerization level. We did however find subfunctionalization at the tetramer level, as the EMSA results showed that ThPI-1 is more strongly engaged in ThSEP3-containing tetrameric complexes than ThPI-2. Other ThSEP proteins however do not differentiate between ThPI-1 and ThPI-2. How relevant this discrepancy between ThPI-1 and ThPI-2 is in higher order complex formation *in planta* depends on the spatiotemporal expression of the different *SEP* genes during flower development, as the expression patterns/levels together with protein-protein affinity will determine the composition of the functional tetrameric MADS domain complexes. According to the floral quartet model [23], a specific tetramer is formed in each type of floral organ, which binds to two adjacent binding sites in regulatory regions of target genes. The composition of the tetramer determines in part the specificity of the protein complex for a particular target sequence. Interestingly, according to RNA-seq data of mature floral organs, the *ThSEP* paralogs are differentially expressed in an organ-specific manner (Additional file 6: Figure S5, data from [72]). Differences in expression of the *ThSEP* paralogs could lead to organ-specific differences in complex formation of *ThPI-1* and *ThPI-2*. Although we show that *ThSEP* genes are differentially expressed in mature floral organs, the expression patterns of the *ThSEP* paralogs during flower development are not yet elucidated.

Another feature that impacts which genes are regulated by a TF is its DNA-binding specificity. We determined DNA-binding specificity of the *T. hassleriana* AP3–1/PI-1 and AP3–1/PI-2 heterodimers in vitro using SELEX-seq, and found slightly different binding motifs for the two AP3–1/PI heterodimers. Both PI/AP3–1 heterodimers bind to CArG-boxes, as expected for MADS-domain proteins. To our knowledge, this is the first determination of DNA-binding specificities of an AP3/PI dimer in any species. The only DNA-binding motif for AP3/PI which is published was generated based on ChIP-seq of the *A. thaliana* AP3/PI heterodimer [93]. However, this motif can also contain information from other MADS proteins that interact with AP3/PI in higher-order complexes. The SELEX-seq based motifs we obtained for the *T. hassleriana* PI paralogs are more similar to each other than to this *A. thaliana* motif, with especially the cytosine at positions 1 and 2 of the CArG-box being more conserved in our *T. hassleriana* motifs than in the published *A. thaliana* motif. However, these differences between the *A. thaliana* motif and our *T. hassleriana* motifs are likely due to differences in methods used to obtain these motifs. SELEX-seq determines the DNA-binding specificity of TFs to unmethylated DNA in vitro. In contrast, ChIP-seq is an in vivo method, where sequences might be bound indirectly, DNA might be methylated and less accessible, and the AP3/PI heterodimer is likely part of a larger protein complex. Both DNA methylation and interaction with cofactors can influence DNA recognition by TFs. Interestingly, we observed subtle differences in specificity between the two *T. hassleriana* ThAP3–1/ThPI heterodimers. This may indicate that these paralogs could regulate partly different targets. It would be interesting to compare DNA-binding properties of B-class heterodimers from a wide range of Brassicales species to pinpoint when these differences originated, and whether they are correlated with the transposition events of *AP3* and *PI*.

That paralogous TFs can exhibit differences in DNA-binding specificity has also been shown for another plant TF, LEAFY (LFY). LFY is an important regulator of floral meristem identity and is present as a single-copy TF in most plant species, with the exception of gymnosperms. Gymnosperms typically have two paralogs, LFY and NEEDLY (NLY). SELEX-seq experiments on these paralogous proteins from *Welwitschia mirabilis* showed that LFY and NLY have different, although overlapping DNA-binding specificities [94]. The differences we observed in DNA-binding specificity between the ThPI paralogs should be experimentally validated. This could be done in vitro, for instance with quantitative EMSAs. To determine whether these differences are relevant in vivo, it would be interesting to perform ChIP-seq experiments with these ThPI paralogs, to determine whether they bind to different sites in the genome.

Although we found differences between the ThPI paralogs in protein-protein interactions and in DNA-binding specificity, we did not investigate whether these differences led to divergence in function in Tarenaya plants. Published data from *PI* duplications in other species show a range of evolutionary possibilities. In some cases, the genes are redundant, as is the case for the petunia and tomato *PI* paralogs. In *Nicotiana benthamiana*, the situation is slightly different as both *PI* genes are necessary for petal and stamen specification [40, 41]. In the Solanaceae species *Physalis floridiana*, as well as in *Medicago truncatula*, the paralogs diverged more substantially, as only one of the *PI* paralogs seems necessary for petal and stamen specification [88, 89, 95, 96]. However, at least for the *Medicago truncatula PI* paralogs, it was shown that they were both still under purifying selection, arguing against one paralog being in the process of becoming a pseudogene [89].

To elucidate how the Th*PI* paralogs evolved in function, functional studies need to be performed in *T. hassleriana*. In the absence of mutants, this type of functional data can be obtained using Virus Induced Gene Silencing (VIGS) or mutants could be generated by transformation with CRISPR/CAS9 constructs. However, protocols for these functional studies first need to be developed for *T. hassleriana*.

## Conclusions

The Cleomaceae species *T. hassleriana* has two copies of the floral B-class gene *PI*. One of these paralogs is located in the same genomic position as the *PI* gene in most angiosperms, but the other copy has transposed into a different genomic location, which it shares with the *PI* genes of Brassicaceae species. These *PI* paralogs have similar spatiotemporal expression patterns, but may have diverged in function as heterologous expression of ThPI-1, but not ThPI-2, led to a phenotype in *Arabidopsis thaliana*. We show that the ThPI paralogs have diverged in interaction specificity, using in vitro methods. EMSAs show that ThPI-1 and ThPI-2 behave differently in protein-complex formation. SELEX-seq experiments using AP3/PI heterodimers show that there may be subtle differences in DNA-binding specificity between ThPI-1 and ThPI-2. Therefore, the PI paralogs of *T. hassleriana* may have diverged from each other in their specificity for both DNA and protein interaction partners.

## Methods

### Plant growth

*Tarenaya hassleriana* seeds were obtained from Eric Schranz, and are the same genotype as the sequenced *Tarenaya* [65]*. Tarenaya hassleriana* was grown in the greenhouse with an average of 22 °C/day and 18 °C/night. *Arabidopsis thaliana col-0* seeds were originally obtained from NASC. *Arabidopsis thaliana* was grown at 20 °C on rockwool under standard long day (18 h/6 h) conditions.

### Synteny plot

Synteny blocks were detected using MCScanX [97]. Related synteny blocks containing *PI* and flanking genes across the Brassicaceae (8 Brassicaceae species+ *T. hassleriana*) and across other angiosperms (12 species+ *T. hassleriana*) were aligned and visualized using one of the python modules of JCVI [98], which is available at https://github.com/tanghaibao/jcvi/wiki/MCscan-(Python-version).

### Alignments and phylogeny

To calculate percentage identities and similarities between the paralogs, http://imed.med.ucm.es/Tools/sias.html was used, with standard settings. Alignments were generated using Muscle [99]. Phylogenetic analyses were performed in Mega6 [100]. Alignments were generated using a codon-based DNA-sequence algorithm. Phylogenies were produced with the Maximum likelihood method and 1000x bootstrap. Mega6 was run with the default settings, which includes the Tamura-Nei model assuming uniform substitution rates as the substitution model. Boxshade was used for the shading of the alignments.

Sequences used were: *Arabidopsis thaliana*, At3G54340 and At5G20240 (TAIR10); *Arabidopsis lyrata*, XM_002877924 And XM_002871885; *Capsella rubella*, Carubv10018833m and Carubv10001962m (genome version 1.0); *Aethionema arabicum*, AA1026G00001 and AA8G00136 (genome version V2.5); *Tarenaya hassleriana*, Th2v17263, Th2v17264, modified Th2v21500 and Th2v23456 (genome version 5); *Gynandropsis gynandra* Ggy15517, Ggy19834 and Ggy29007 (genome version V3, unpublished); *Carica papaya*, EF562500; *Theobroma cacao*, XM_007017619 and XM_007019158; *Populus trichocarpa*, XM_002300928 and XM_002307424; *Vitis vinifera*, EF418603 and NM_001280946.

### RNA isolation and cDNA synthesis

RNA was isolated from *T. hassleriana* inflorescences using the RNeasy plant mini kit (Qiagen) according to the manufacturer’s instructions, followed by DNAse treatment (Turbo DNA-free, Ambion). cDNA was made using the RevertAid H Minus first strand cDNA synthesis kit (Fermentas) and a custom primer (5″GGCCAGGCGTCGACTAGTACTTTTTTTTTTTTTTTTT 3″).

### RNA in situ hybridisation

3’RACE was used to determine the sequence of the 3’UTR. Fragments were obtained by PCR using a 3’RACE primer (GGCCACGCGTCGACTAGTAC) and a gene-specific primer, followed by a PCR with a nested gene-specific primer (primers see Table 1). The obtained fragments were cloned into PCR®2.1 TOPO® (ThermoFisherScientific) and sequenced.

**Table 1:** Primers used for the 3’RACE and RNA in situ hybridisation. Primers to obtain the 3’UTR sequence as well as to generate the in situ RNA probes are shown

In situ probes	Fw	R
*ThAP3*	CTCTCCATTCTCTGCGACGCTAG	CATCAAGCTAGGTTTTTCAACTCC
*ThPI-1*	GCTCTCCTTCAATGGATCTTGGTG	CACTTATGTCCAAGTCCTTGCAGAG
*ThPI-2*	GATCACTGTTCTATGCGACGCC	GAAACACGCAACGAACCTTGTC
3’ RACE	First PCR	Nested PCR
*ThAP3–1*	CTCACTACGAAAGGATGCAAGAGAC	GAAGTTTAAATCGATTGGCAGCC
*ThAP3–2*	CCTCTCACTACGAAAGGATGCAG	CGATTGGCAATAAAATTGAAACC
*ThPI-1*	GAGCAGTATCAAAGGATCGCC	GGCCATAGAGCACGCAGTCC
*ThPI-2*	GAGATGTTGGGCACTTATCAGC	CAAAAGCCTAATCGCCATAGAGAG

RNA in situ hybridisation was performed as described in [101]. Sequences downstream of the MADS-domain coding sequence were used as probes (primers used can be found in Table 1). These sequences were cloned into PCR2.1® TOPO® (ThermoFischer Scientific) under the T7 promoter and used to prepare digoxigenin-labelled RNA probes. Pictures were taken with a Leica DM6000 microscope and processed with Fiji (ImageJ).

### EMSAs

*T. hassleriana* genes were amplified from cDNA and cloned into pSPUTK (primers shown in Table 2). Proteins were synthesized using the TnT® SP6 High-Yield Wheat Germ Protein Expression System (Promega) according to the manufactures instructions, using a total of 60 ng plasmid/μl reaction. Proteins for interaction assays were always co-translated, using equimolar amounts of the different plasmids. EMSAs were performed as described in [74] with minor modifications. The fluorescent dye DY-682 was used to label the oligonucleotides. Labelled oligonucleotides were produced by PCR using vector-specific DY-682-labelled primers, and purified from agarose gel. The binding mix was modified by replacing the glycerol with loading dye. This modification changed the concentration EDTA from 1.2 to 2.2 mM, and added 1 mM Tris-Hcl (pH 7.5), 6.5% sucrose and 0.03% Orange G. For the higher-order complexes, a 4.75% gel was used. Gel-shifts were visualized with the LiCor Odyssey at 700 nm. For EMSA experiments, we used a previously published probe from an *AthSEP3* upstream enhancer element [74] (pGEM-T sequence underlined): 5′CATGGCCGCGGGATTTTGACGATAACTCCATCTTTCTATTTTGGGTAACGAGGTCCCCTTCCCATTACGTCTTGACGTGGACCCTGTCCGTCTATTTTTAGCAGAATCACTAGTGCGGCCGC-3′.

**Table 2:** Primers used to generate pSPUTK constructs used for in vitro protein production

pSPUTK cloning		
Gene	Fw	R
*AP3–1/2*	GATAGATCTATGACGAGGGGAAAGATTCAG	GATAGATCTTCATTCGAGCAAGTGGAAGG
*ThPI-1*	TTACCATGGGGAGAGGAAAGATAGAG	ATTATCGATCAGTCGATGACCAAAGACATGATC
*ThPI-2*	AATCCATGGGAAGAGGGAAGATAGAGATCAAAAG	TTTATCGATCAGACGATGTGTTGTAAATTGGGC
*Th2954* (*AG*)	ACGGCGTACCAAACGGAGTTG	TTACACTAACTGAAGTGGAGTGTG
*Th1528* (*SEP3*)	CATGCCATGGGAAGAGGTCGTGTTGAG	AAGATCGATCAATTGTTGTCATAAGGTAACCAAC
*Th18678* (*SEP3*)	CATGCCATGGGGAGAGGTCGAGTTG	AAGATCGATCAATTGTTGTCGTAAGGTAACCAAC
*Th21984* (*SEP4*)	ATGGGAAGAGGGAAAGTGGAGC	TCAGATCATCCAGCCGTGGAA
*Th2854* (*SEP1/2*)	ATGGGGAGGGGTAGGGTTG	TCAGAGCATCCAACCAGGG
*Th13754* (*AP1*)	ATGGGAAGGGGAAGGGTTCAG	TTATGTGAAGCAGCCAAGGTTGCAATC

### Overexpression

The ThPI-1 and ThPI-2 protein coding sequences were amplified (primers in Table 3) and ligated into gateway vector pCR8 (Thermo Fisher Scientific). Subsequently the sequences were transferred via LR reaction into the destination vector pB7WG2, a binary vector for 35S-based overexpression in plants [102]. Constructs were transformed into *A. thaliana Col*-0 by the floral dip method [103]. Plants were selected on ½ Murashige-Skoog (MS) media 0.8% agar plates containing 10 μl/ml PPT. 12–14 independent transgenic lines were analysed in the T1 generation.

**Table 3:** Primers used for heterologous expression experiment

	F	R
*ATPI*	ATGGGTAGAGGAAAGATCGAG	TCAATCGATGACCAAAGACATAATC
*Th* *PI-1*	ATGGGGAGAGGAAAGATAGAG	TCAGTCGATGACCAAAGACATG
*ThPI-2*	ATGGGAAGAGGGAAGATAGAG	TCAGACGATGTGTTGTAAATTGG
qPCR *AtPI*	GATCATGATGGGCAGTTTGGATATAG	TCGATGACCAAAGACATAATCTTTTCC
qPCR *ThPI-1*	GACATCCAGTCCCTGGACATC	TCCGTTTCTACGTTTCGTC
qPCR *ThPI-2*	GACATCCAATCTATGAACTAT	CTCCCTTCTCTTCAATTCT
qPCR *TIP41* (reference)	GTGAAAACTGTTGGAGAGAAGCAA	TCAACTGGATACCCTTTCGCA

Total RNA was prepared from leaves of all transgenic lines using the Invitrap spin plant RNA mini kit (Stratec) according to the manufacturer’s instructions. cDNA was prepared using the iScript cDNA synthesis kit from Biorad, according to the manufacturer’s instructions. Expression levels were determined by RT-qPCR. Expression level was measured in leaves of 6–8 week old plants, and normalized against *TIP41* expression (*At4g34270*) [104] (primers used are in Table 3).

### SELEX-seq

SELEX-seq was essentially performed a described before [105]. The dsDNA libraries contained 40-nucleotide randomized sequences flanked by specific barcodes that allowed for multiplexing in high-throughput sequencing. The dsDNA libraries contained all necessary features required for direct sequencing with an Illumina Genome Analyzer [81]. Proteins were synthesized using the TNT SP6 Quick Coupled Transcription/Translation System (Promega) following the manufacturer’s instructions in a total volume of 20 μl. The binding reaction mix was prepared essentially as described previously for EMSA experiments [74] and contained 20 μl of in vitro-synthesized proteins and 50–100 ng of dsDNA in a total volume of 120 μl. The binding reaction was incubated for 1 h at 21 °C followed by 1 h immunoprecipitation with 20 μl anti-HA antibodies coupled to magnetic beads (ThermoScientific) in a thermomixer at 21 °C with constant mixing at 700 rpm. After immunoprecipitation, beads were washed 5 times with 150 μl binding buffer without salmon-sperm DNA, rinsed once with 500 μl of 1xTE and bound DNA was eluted with 50 μl 1X TE by incubation in a thermomixer for 20 min at 90 °C at full mixing speed. Following this incubation, magnetic beads were immobilized and the supernatant containing the eluted DNA was transferred to a new tube. DNA fragments were amplified for 5 to 11 cycles of PCR with SELEX round-specific primers [81] and the total amplicon was used in the subsequent SELEX round. The amplification efficiency was checked on an agarose gel. Samples for sequencing were amplified, size-selected by agarose gel purification using the Qiaquick Gel Extraction Kit (Qiagen). Different libraries were multiplexed by mixing equimolar amounts, and sequencing was performed on the HiSeq 2000 (Illumina). Data was analyzed as in [105], namely sequence reads that did not pass the filter quality of CASAVA or mapped with no mismatches to the phix174 genome were eliminated. Then we calculated frequencies of the k-mer sequences in each Round except Round 0. Sequences in Round 0 represent a set of randomly synthetized oligonucleotides and their complexity did not allow for the direct calculation of k-mer frequencies. Therefore, the sequence frequency in Round 0 was estimated by the sixth-order Monte Carlo model, as proposed before [106]. Relative affinity for each possible k-mer was calculated as the ratio between the frequencies of k-mers in final Round of enrichment to Round 0, and normalized to 1 by dividing for the highest affinity predicted k-mer.

## Additional files

Additional file 1: **Figure S1.** DNA coding sequence alignment of *T. hassleriana* AP3 paralogs. Both coding sequence and 3’ UTR are shown. # indicates the start codon, whereas the * indicates the stop codon. (PNG 121 kb)

Additional file 2: **Table S1.** Estimates of the isoelectric point of the four *T. hassleriana* B-class proteins. (DOCX 13 kb)

Additional file 3: **Figure S2.** EMSAs to test for higher order complexes containing AP3/PI heterodimers. **(A)** Combinations of each of the four heterodimers with AG and one of the two SEP3 paralogs (Th1528 on the left, Th18678 on the right). The two SEP3 paralogs gave similar results. We studied the interaction of AG and one of the SEP3 paralogs (Th1528) and the B-class heterodimers in more detail for AP3–1/PI-1 **(B)** and AP3–1/P-2 **(C)** (see also Fig. 3b). (PNG 212 kb)

Additional file 4: **Figure S3.** *Arabidopsis thaliana* anti-AP3 antibody (AB) recognizes the *Tarenaya hassleriana* AP3 paralogs. Recognition of all four B-class heterodimers by the *A. thaliana* AP3 antibody was assessed using EMSA. For each heterodimer, a supershift of the complex is observed when the AB is added (right) compared to the no AB control (left). (PNG 157 kb)

Additional file 5: **Figure S4.** DNA-binding specificities as determined by SELEX-seq. EMSAs showing enrichment of bound sequences in different SELEX rounds of the ThPI-1/ThAP3–1 (**A**) and ThPI-2/ThAP3–1 (**B**). Round that is sequenced is indicated in red. (PNG 214 kb)

Additional file 6: **Figure S5.** SEP paralog expression in mature flowers based on RNA-seq data. Th21984, (a SEP4 paralog) was not present in the dataset. The other SEP4 paralog is hardly expressed. SEP3 and SEP1/2 are both expressed, but expression levels differ between paralogs and between organs. Data from [72]. (PNG 54 kb)
