# Supplementary figures and images for: PISTILLATA paralogs in Tarenaya hassleriana have diverged in interaction specificity
Source: BMC Plant Biol. 2018 Dec 22;18:368. doi: 10.1186/s12870-018-1574-0 (PMC6303913; doi:10.1186/s12870-018-1574-0)

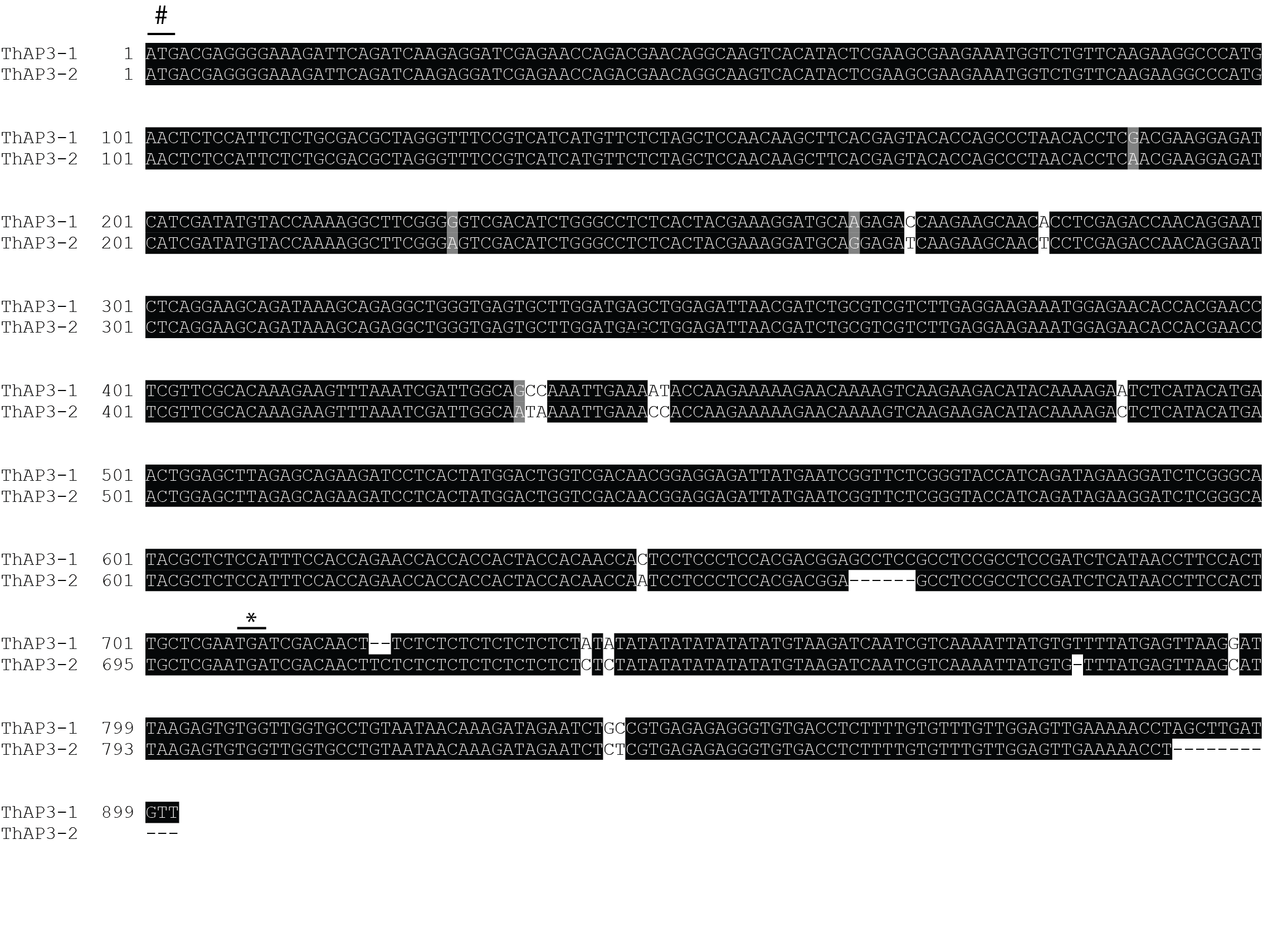

Supplement: Supplementary file 1 — Figure S1. DNA coding sequence alignment of T. hassleriana AP3 paralogs. Both coding sequence and 3’ UTR are shown. # indicates the start codon, whereas the * indicates the stop codon. (PNG 121 kb) [file 12870_2018_1574_MOESM1_ESM.png]

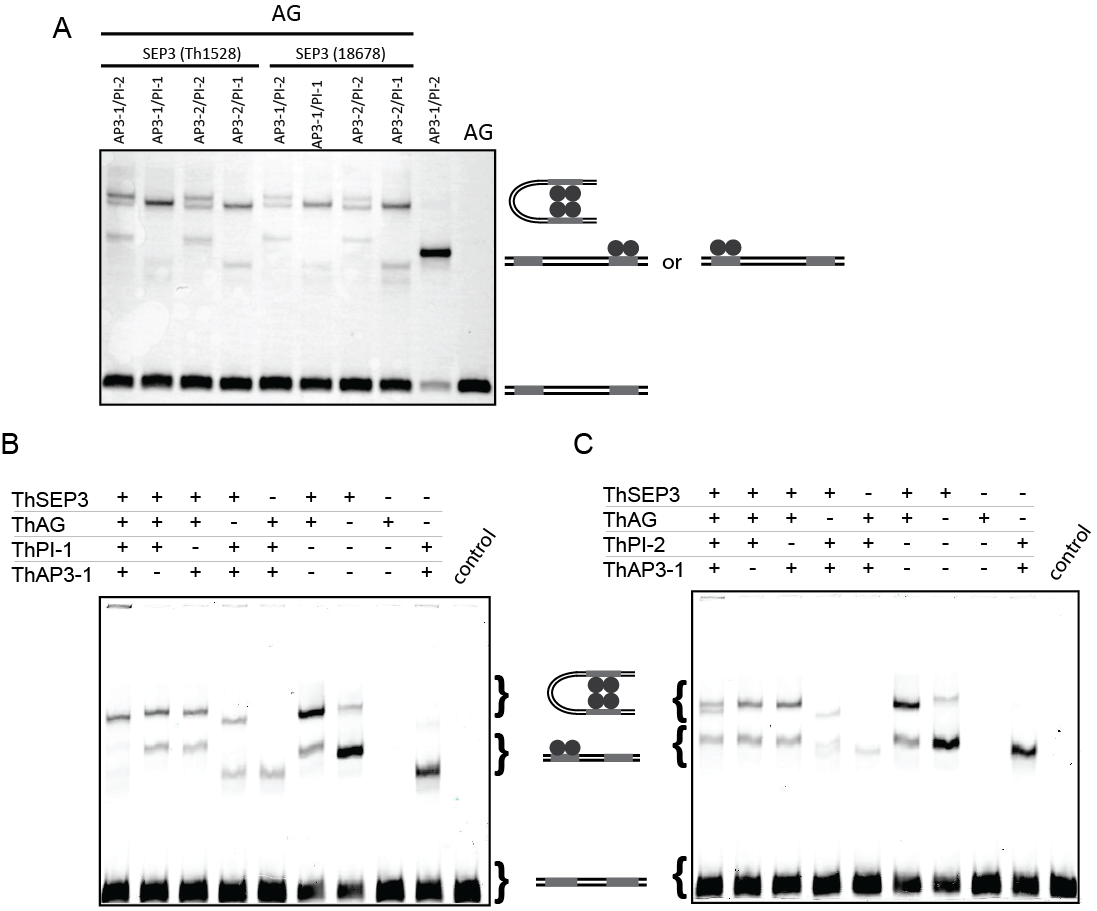

Supplement: Supplementary file 3 — Figure S2. EMSAs to test for higher order complexes containing AP3/PI heterodimers. (A) Combinations of each of the four heterodimers with AG and one of the two SEP3 paralogs (Th1528 on the left, Th18678 on the right). The two SEP3 paralogs gave similar results. We studied the interaction of AG and one of the SEP3 paralogs (Th1528) and the B-class heterodimers in more detail for AP3–1/PI-1 (B) and AP3–1/P-2 (C) (see also Fig. 3b). (PNG 212 kb) [file 12870_2018_1574_MOESM3_ESM.png]

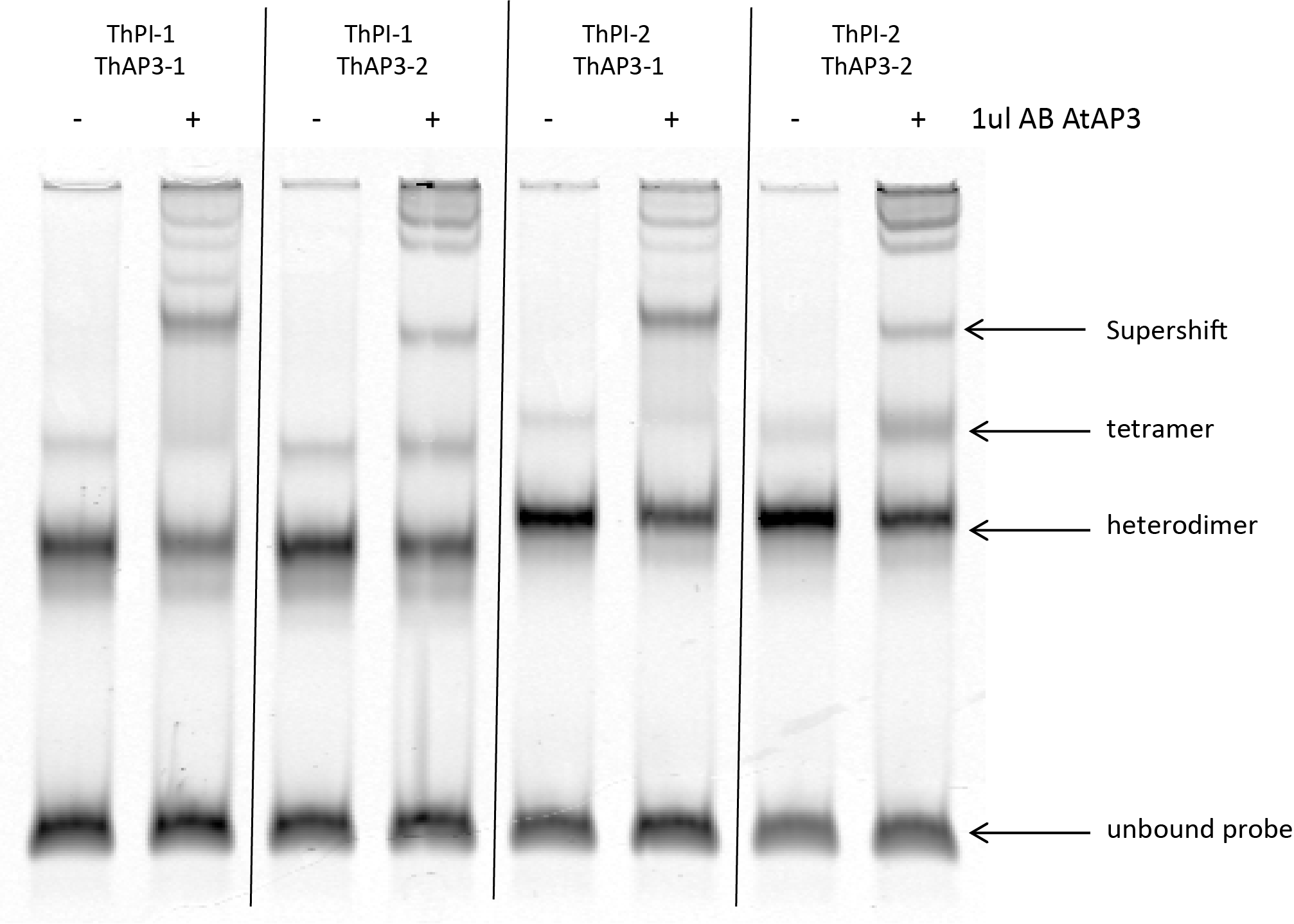

Supplement: Supplementary file 4 — Figure S3. Arabidopsis thaliana anti-AP3 antibody (AB) recognizes the Tarenaya hassleriana AP3 paralogs. Recognition of all four B-class heterodimers by the A. thaliana AP3 antibody was assessed using EMSA. For each heterodimer, a supershift of the complex is observed when the AB is added (right) compared to the no AB control (left). (PNG 157 kb) [file 12870_2018_1574_MOESM4_ESM.png]

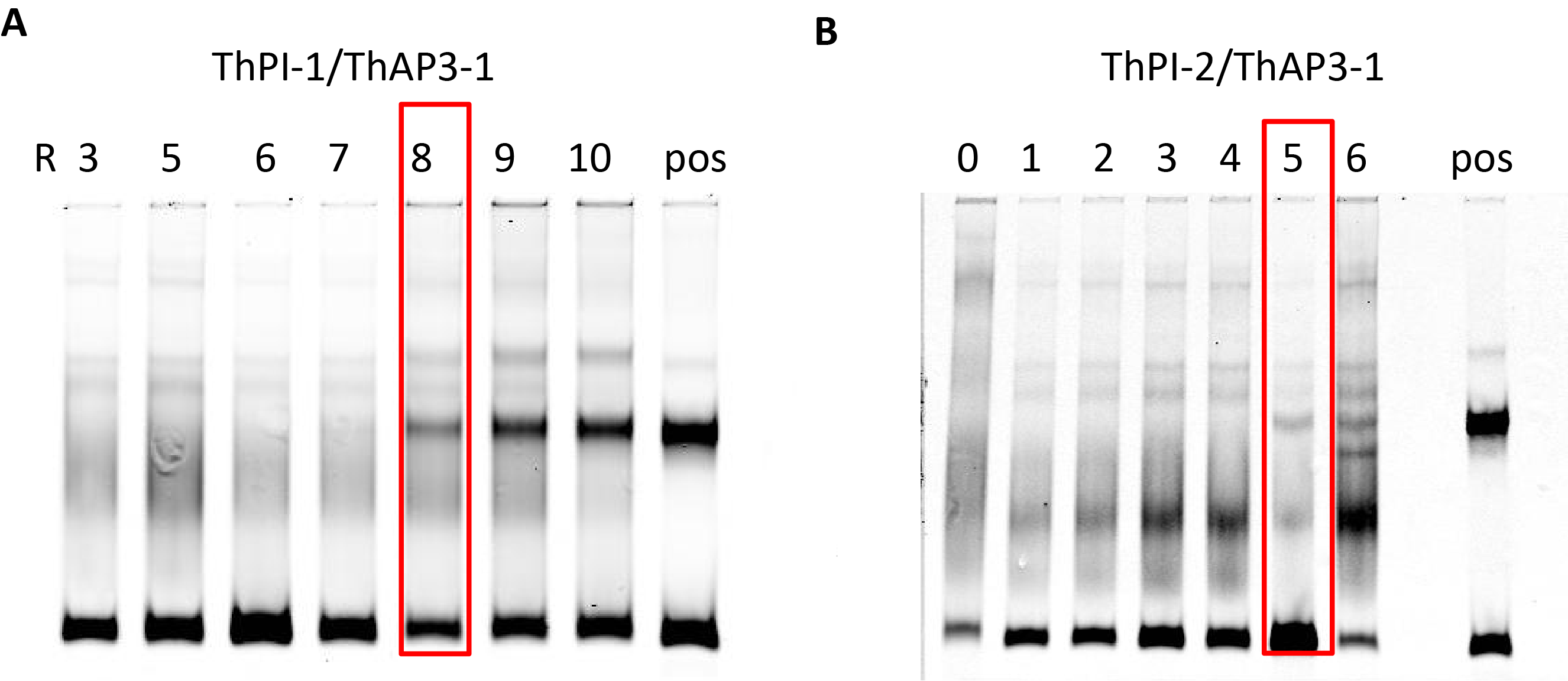

Supplement: Supplementary file 5 — Figure S4. DNA-binding specificities as determined by SELEX-seq. EMSAs showing enrichment of bound sequences in different SELEX rounds of the ThPI-1/ThAP3–1 (A) and ThPI-2/ThAP3–1 (B). Round that is sequenced is indicated in red. (PNG 214 kb) [file 12870_2018_1574_MOESM5_ESM.png]

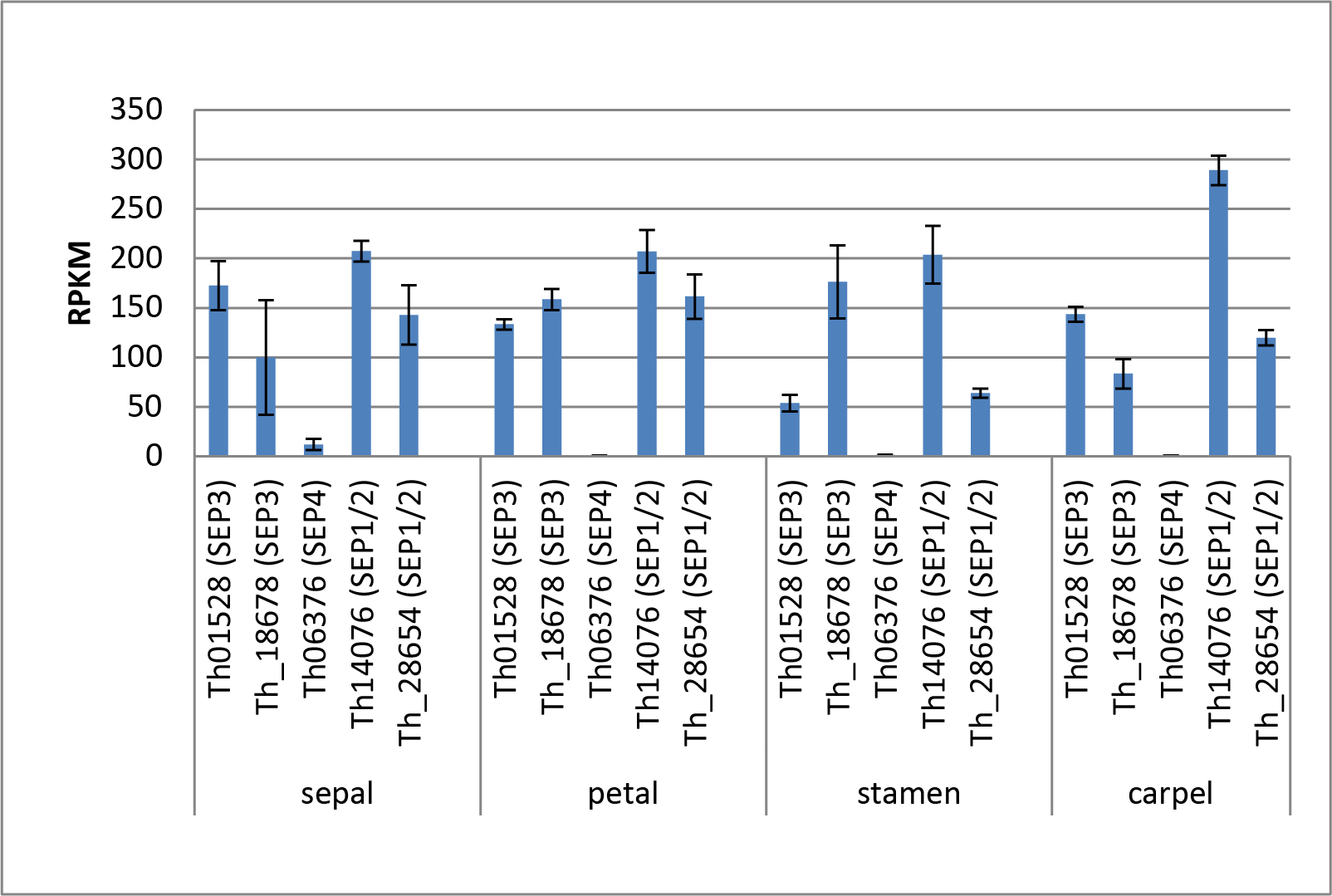

Supplement: Supplementary file 6 — Figure S5. SEP paralog expression in mature flowers based on RNA-seq data. Th21984, (a SEP4 paralog) was not present in the dataset. The other SEP4 paralog is hardly expressed. SEP3 and SEP1/2 are both expressed, but expression levels differ between paralogs and between organs. Data from [72]. (PNG 54 kb) [file 12870_2018_1574_MOESM6_ESM.png]
