# Supplementary material for: PISTILLATA paralogs in Tarenaya hassleriana have diverged in interaction specificity
Source: BMC Plant Biol. 2018 Dec 22;18:368. doi: 10.1186/s12870-018-1574-0 (PMC6303913; doi:10.1186/s12870-018-1574-0)
Supplement: Supplementary file 2 — Table S1. Estimates of the isoelectric point of the four T. hassleriana B-class proteins. (DOCX 13 kb) [file 12870_2018_1574_MOESM2_ESM.docx]

**Table S1: Estimates of the isoelectric point of the four *T. hassleriana* B-class proteins.**

|  | **http://pepcalc.com/** | | **http://isoelectric.ovh.org/calculate.php** | **Expasy compute PI/MW** |
| --- | --- | --- | --- | --- |
|  | **Ph 7 charge** | **Isoelectric point** | **Isoelectric point** | **Isoelectric point** |
| **ThAP3-1** | **3.5** | **8.54** | **7.73** | **8.39** |
| **ThAP3-2** | **4.3** | **8.91** | **7.96** | **8.69** |
| **ThPI-1** | **4.4** | **9.14** | **8.04** | **8.93** |
| **ThPI-2** | **5.2** | **9.12** | **8.12** | **9.01** |
